# Evaluation of the French surveillance system for epidemiological surveillance of antimicrobial resistance in the community and nursing homes

**DOI:** 10.1093/jacamr/dlac078

**Published:** 2022-07-04

**Authors:** Lucie Collineau, Euriel Godebert, Sonia Thibaut, Olivier Lemenand, Gabriel Birgand, Jocelyne Caillon, Clémence Bourely

**Affiliations:** French Agency for Food, Environmental and Occupational Health & Safety, Laboratory of Lyon, Epidemiology and surveillance Unit, Lyon, France; French Agency for Food, Environmental and Occupational Health & Safety, Laboratory of Lyon, Epidemiology and surveillance Unit, Lyon, France; French National School of Veterinary Services (VetAgro Sup—ENSV), Marcy-l’Étoile, France; French Ministry of Agriculture and Food, Directorate General for Food, Animal Health Unit, Paris, France; Regional centre for prevention of healthcare-associated infections (CPias), University Hospital of Nantes, Nantes, France; Regional centre for prevention of healthcare-associated infections (CPias), University Hospital of Nantes, Nantes, France; Regional centre for prevention of healthcare-associated infections (CPias), University Hospital of Nantes, Nantes, France; Regional centre for prevention of healthcare-associated infections (CPias), University Hospital of Nantes, Nantes, France; French Ministry of Agriculture and Food, Directorate General for Food, Animal Health Unit, Paris, France

## Abstract

**Background:**

Antimicrobial resistance (AMR) has been widely recognized as a major public health issue, which can be addressed through effective AMR surveillance systems. In 2018, a national surveillance programme for AMR in the community and nursing homes called Mission PRIMO was established in France. It builds on an existing network called MedQual-Ville that had been monitoring AMR mainly in the west of France community since 2003.

**Objectives and Methods:**

To evaluate the MedQual-Ville surveillance activities and to formulate practical recommendations for improvement, using a semi-quantitative evaluation framework called OASIS.

**Results:**

The evaluation showed that MedQual-Ville is overall a well-performing surveillance system. Its major strengths rely on excellent coordination and internal communication with clinical laboratories that participate on a voluntary basis. Surveillance objectives and procedures are clear to all participants. Hence, the quality and reliability of the data being produced is very high. At this stage, the major area for improvement is representativeness, with poor coverage achieved in several densely populated areas. Besides, the utility and impact of surveillance data could be improved by strengthening communication towards end-users, especially local prescribers.

**Conclusions:**

There is currently no European programme or guidance for AMR surveillance in the community and nursing homes. Our results partly fill this gap, by evaluating how surveillance is being performed in France and providing recommendations that could be applicable to other countries with similar health systems. This work also highlighted the relevance of OASIS for evaluation of surveillance systems in the human sector.

## Introduction

Antimicrobial resistance (AMR) has been widely recognized as a major public health issue and is estimated to account for approximately 670 000 infections and 33 000 deaths in the European Union and European Economic Area in 2015.^1^ In France, these were estimated to be approximately 125 000 infections and 5500 deaths, respectively.^1^ The associated healthcare costs are substantial and were estimated to be EUR 109.3 million in France in 2015, hence representing a major economic burden.^2^ One way to address this issue is through strong and effective AMR surveillance systems, which are essential to describe trends, determine how antibiotic consumption and AMR are linked, detect events, set priorities and evaluate policies.^3^

In 2018, the French Public Health Agency (Santé Publique France; SpF), supported by a specific expert committee, established five national surveillance and prevention programmes on healthcare-associated infections, antibiotic consumption and AMR in hospitals, the community and nursing homes. These programmes have been delegated to six regional centres for prevention and control of healthcare-associated infections (called CPias). More specifically, the CPias of the region Pays de la Loire in collaboration with the CPias of the region Grand-Est is in charge, for the period 2018–23, of the national programme called Mission PRIMO, which focuses on surveillance and prevention of AMR and healthcare-associated infections in the community and nursing homes.^4,5^ While Mission PRIMO was launched only in 2018, it built on a previously existing network of clinical laboratories called MedQual-Ville, which had been collecting AMR surveillance data in the community mainly in western France since 2003.^6,7^ However, surveillance activities performed by MedQual-Ville have not been thoroughly evaluated so far. More generally, no European or international guidelines on AMR surveillance in the community currently exist, and previous literature providing best-practice recommendations is rather scarce.^8–10^

A wide range of surveillance evaluation frameworks has been proposed.^11,12^ Among others, the OASIS semi-quantitative framework was developed by the French Agency for Food, Environmental and Occupational Health & Safety (ANSES) to support thorough evaluations of surveillance systems, initially in the animal and food sectors.^13^ OASIS has been applied to multiple surveillance systems in France, including a recent application to the AMR surveillance system in animal pathogens called RESAPATH, an organization that is very similar to MedQual-Ville.^14^ However, to our knowledge, OASIS has never been applied to a surveillance system in the human sector. ANSES is currently revising this tool to make it more generic and applicable to any surveillance system, including in the human sector.

Hence, the objectives of the present study were (i) to evaluate the French system for AMR surveillance in the community and nursing homes; (ii) to formulate practical recommendations for improvement; and (iii) to assess opportunities and challenges for applying the OASIS evaluation framework to a surveillance system in the human sector.

## Methods

### The MedQual-Ville surveillance system

The MedQual-Ville surveillance system involves a growing network of clinical laboratories located across metropolitan France and participating on a voluntary basis (742 laboratory sites in 2018 and 1016 in 2019, i.e. a participation rate of 18.3% and 24.9% in 2018 and 2019, respectively).^15,16^ Upon signature of a membership charter, participating laboratories commit to send all their results of antimicrobial susceptibility testing (AST) performed on *Escherichia coli*, *Enterobacter* spp., *Klebsiella* spp. and *Staphylococcus aureus* isolated for diagnostic purposes from primary healthcare and nursing homes patients. In 2018, 451 183 antibiograms were collected by MedQual-Ville, out of which 93.4% were Enterobacterales strains isolated from urine samples with 90.4% being *E. coli.*^15^

Data are sent by clinical laboratories either monthly, quarterly or twice-yearly.^17^ Collected raw data include identification of the strain, sampling date, type of specimen, laboratory location, bacteria species isolated, AMR profile (susceptible, intermediate or resistant) and AMR phenotypes (production of ESBLs, carbapenemases and/or cephalosporinases), as well as patient housing type (home or nursing home), sex and age. The data collection protocol is approved by the French data protection authority (CNIL). In addition, MedQual-Ville performs an annual survey among all participating laboratories to describe the methodology they use, including laboratory techniques, standards and interpretation criteria, as well as data management tools and practices. AST techniques vary between laboratories (including disc diffusion or microdilution, with or without automation), but they are all considered as equivalent by MedQual-Ville as they have been validated by the AMR national reference laboratories (NRL). On the other hand, AST standards and interpretation criteria are harmonized and all laboratories follow the recommendations of the Antibiogram Committee of the French Society of Microbiology (CA-SFM),^18^ which are in line with EUCAST.

Upon data reception by the MedQual-Ville coordination team, data are cleaned and duplicates removed via an automated script written in SAS® software, which has been developed by MedQual-Ville specifically for each participating laboratory, in order to accommodate differences in data formats and laboratory information management systems (LIMS). Next, data go through a validation algorithm to check for agreement with the CA-SFM/EUCAST consistency rules. Validated data are integrated within a MySQL database, while non-validated data (approx. 4% in 2018) are placed in quarantine and checked manually prior to inclusion in the database. Descriptive data analysis is performed by geographical department and region, as well as patient sex, age and housing type. Indicators of interest include, for each combination of bacteria species and antimicrobial agent, the proportion of resistant strains out of all strains received. In addition, the proportions of specific phenotypes, including MRSA and ESBL-, cephalosporinase- and carbapenemase-producing Enterobacterales are being monitored.^15^ Quarterly, twice-yearly and annual reports are produced and disseminated to a wide audience, including the participating laboratories, the 17 CPias and SpF. Data visualization is also provided via the online open-access tools MedQual-Ville and Géodes.^17,19^

### Description of the OASIS evaluation framework

**Figure 1.**
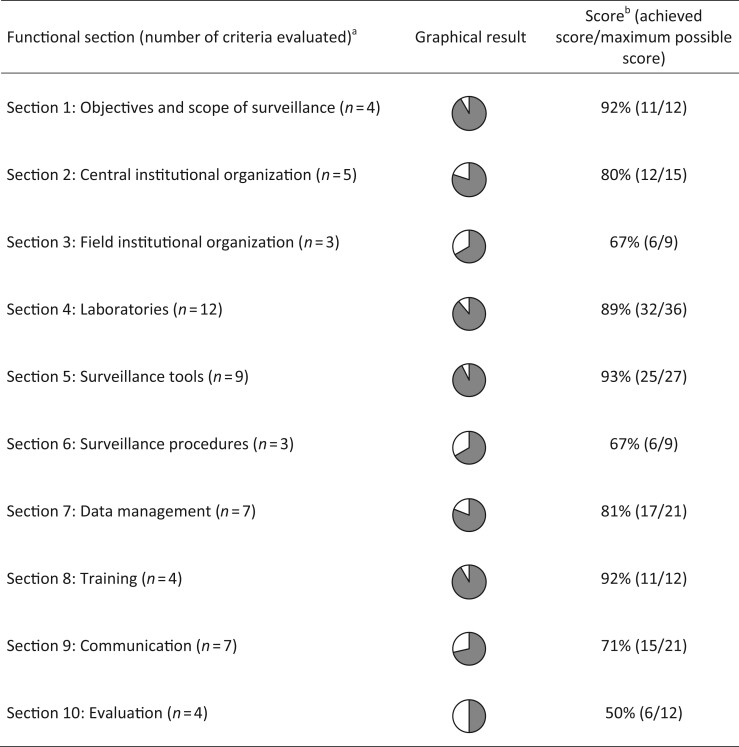
Scores of MedQual-Ville surveillance obtained for the 10 OASIS functional sections (Output 1). ^a^ Criteria considered as not applicable were excluded here. ^b^ Score is expressed as the percentage of the maximum achievable score.

The OASIS evaluation framework is a semi-quantitative and generic approach for the evaluation of health surveillance systems.^13^ It relies on a scoring grid developed in a Microsoft Excel spreadsheet that includes 78 assessment criteria articulated around 10 sections that cover the key aspects of the surveillance process, including: (i) objectives and scope of surveillance; (ii) central institutional organization; (iii) field institutional organization; (iv) diagnostic laboratories; (v) surveillance tools; (vi) surveillance procedures; (vii) data management; (viii) training; (ix) results dissemination; and (x) evaluation and performance (Figure 1 and Table S1, available as Supplementary data at *JAC-AMR* Online). A score from 0 (minimum score) to 3 (maximum score) is attributed to each criterion with support from a scoring guide, according to the level of compliance of the system under evaluation in comparison with an ‘ideal’ surveillance system. Justifications for each score are also provided in a comments box and form the basis for the formulation of practical recommendations for improvement. A criterion can be rated as ‘not applicable’ if not relevant to the surveillance system under evaluation; in this case, the criterion has no impact on the evaluation results. Data collection required to inform the scoring grid is facilitated by a 40-page questionnaire organized around the 10 sections mentioned above. The questionnaire is completed using available literature (including annual reports, scientific publications, protocols, etc.), as well as semi-directed interviews with key actors and end users of the system.

**Figure 2.**
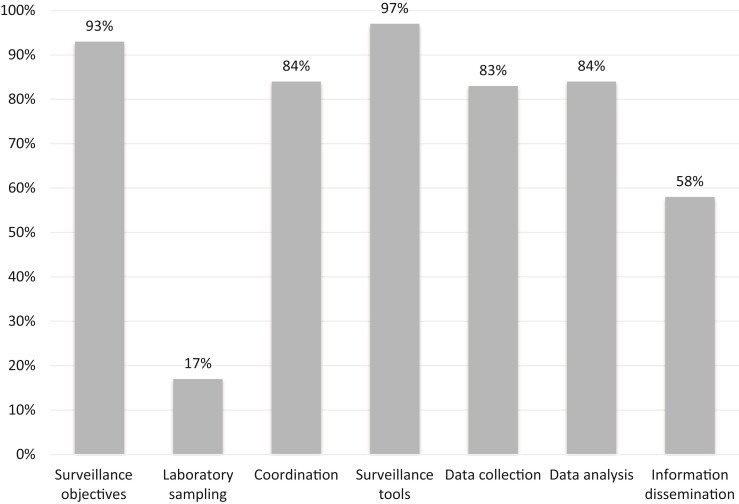
Scores of MedQual-Ville surveillance obtained for the seven OASIS CCPs (Output 2). Scores indicate the percentage of the maximum achievable score.

**Figure 3.**
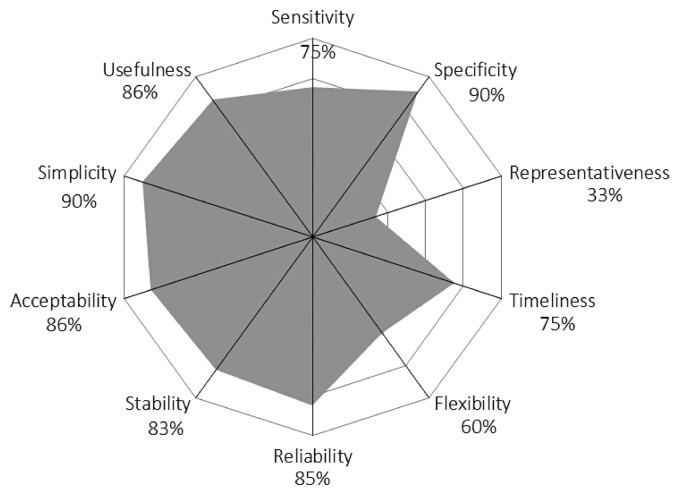
Scores of MedQual-Ville surveillance obtained for the 10 OASIS attributes (Output 3). Scores indicate the percentage of the maximum achievable score.

Upon completion of the scoring grid, evaluation results are displayed using three outputs that provide a complementary view of the surveillance system: (i) Output 1 displays results by 10 functional sections and provides a general view of the structure and operations; it enables the weak functional parts of the system to be identified easily (Figure 1); (ii) Output 2 is derived from a critical control point (CCP) approach and aims to identify the level of control of seven CCPs of the surveillance system (Figure 2); this output is particularly useful for proposing operational improvements; and (iii) Output 3 displays the evaluation results by surveillance attributes as defined by the US CDC and WHO (Figure 3)^20,21^; it highlights the effectiveness attributes of the system for which improvement is needed. Of note, the assessment criteria are weighted in Outputs 2 and 3, while they are not in Output 1. Calculation details for each output are provided in Table S1.

### Application of OASIS to the MedQual-Ville surveillance system

Upon formalization of the request for evaluation by the Mission PRIMO responsible person, an evaluation team was built including three external evaluators (not involved in MedQual-Ville activities but familiar with the OASIS framework) and two internal evaluators (part of the MedQual-Ville coordination team). The inclusion of internal evaluators is typical of the OASIS approach, which aims to be participatory, in order to improve the relevance and future implementation of the recommendations provided. In that sense, OASIS differs from other audits or external evaluations.

The OASIS questionnaire was completed with data collected between March and June 2020, from available literature as well as semi-directed interviews with 26 actors and end users of the system (Table 1). Interviews were organized by telephone because of the COVID-19 travel restrictions and lasted between 40 min and 2 h per interview (1.25 h on average). Interviews were not recorded but evaluators shared their notes in order to obtain a detailed and consolidated written report of each interview. Interviewees were informed that their statements were treated anonymously and reported in an aggregated format, so they could freely express their views, including expectations, perceived strengths and weaknesses of the surveillance system, as well as possible solutions for improvement.

**Table 1.** Profile of interviewees and one-day workshop participants

Interviewees (*n* = 26)	One-day workshop participants (*n* = 13)
All members of the MedQual-Ville coordination team (*n* = 7)	All members of the evaluation team (*n* = 5)
Representatives^a^ of clinical laboratories participating in MedQual-Ville (*n* = 5)	Other members of the Mission PRIMO (*n* = 3)
GPs with regional or national duties to promote antimicrobial stewardship in primary healthcare (*n* = 5)	Representatives of clinical laboratories participating in MedQual-Ville (*n* = 2)
Representatives of CPias other than CPias Pays de la Loire (*n* = 4)	Representative of the NRL for AMR in Enterobacterales (*n* = 1)
Representatives of the NRL for AMR in Enterobacterales and *Staphylococcus* spp. (*n* = 2)	Representative of the CPias Grand Est in charge of the SPARES programme (*n* = 1)
Representative of the French Ministry of Health (*n* = 1)	GP (*n* = 1)
Representative from SpF (*n* = 1)	
Representative from the expert group^b^ in charge of the annual evaluation of the AMR national missions, including Mission PRIMO (*n* = 1)	

a Representativeness was maximized in terms of geographical regions and data volumes.

b The evaluation performed by this expert group is an evaluation of the Mission PRIMO as a whole (including AMR surveillance and other activities, e.g. prevention of healthcare-associated infections) and goes beyond the scope of this evaluation.

Building from the questionnaire, a first version of the scoring grid, including justification of each score, was completed by the evaluation team. Then, the pre-completed grid was discussed in detail criterion by criterion during a one-day workshop held in a mixed online and in-person format with 13 participants including both actors and end users of the system (Table 1). Where needed, scores and comments were edited by consensus. The evaluation protocol was internally reviewed by the ANSES Department of Legal Affairs and performed in agreement with the European and French regulations on personal data protection (GDPR).

## Results

The three outputs of the OASIS evaluation are displayed in Figures 1, 2 and 3, respectively. Twenty out of 78 criteria of the OASIS framework were considered as not applicable to the system under evaluation and were not scored (see Table S1). Overall, the MedQual-Ville surveillance system obtained high OASIS scores. The surveillance objectives were clearly defined and formalized in a surveillance protocol (Figures 1 and 2). The expectations of the majority of MedQual-Ville partners were taken into account when defining the surveillance objectives and corresponding activities. At central level, the coordination team had a clear and active role in the MedQual-Ville surveillance system, including the recruitment of participating laboratories, data management and analysis, and dissemination of the results (Figures 1 and 2). Of note, some activities were being conducted informally (e.g. laboratory recruitment) and did not appear in the MedQual-Ville activity report; hence, resources dedicated to these activities were likely underestimated. The MedQual-Ville coordination team was rather small (2.5 full-time equivalents), which could be an issue should replacement be needed. Conversely, material and financial resources generally appeared sufficient to cover current operating costs. The coordination team was supported by a scientific committee specific to MedQual-Ville that included all relevant partners and disciplines. Conversely, the steering committee (which decides on the vision and orientations of the overall Mission PRIMO) was joint together with the four other national missions on healthcare-associated infections; hence, a few MedQual-Ville key partners (e.g. clinical laboratories, NRL or GPs) were not represented.

Clinical laboratories were fully integrated into the surveillance system (Figure 1). Written guidance documents were provided by the MedQual-Ville coordination team, and individual follow-up was provided for additional assistance, e.g. to facilitate the first data extraction and data submission. Clinical laboratories had data ownership and access at any time, but MedQual-Ville could use their data freely. Laboratories were not financially compensated for their time dedicated to data extraction, upload and validation. While these activities may be perceived as time-consuming, interviewees reported that time involved was limited (15 min to 2 h monthly, depending on the level of automation) compared with the benefits of their participation (e.g. benchmarking of AMR data at regional/departmental levels, free external data quality check, contribution to an initiative of public health importance). Sampling procedures were very simple (primarily non-invasive urine samples), and results were delivered in a timely manner (Figure 3). AST sensitivity and specificity were very high (Figure 3), and AST was accredited in 100% of participating laboratories, ensuring high level of harmonization.

**Figure 4.**
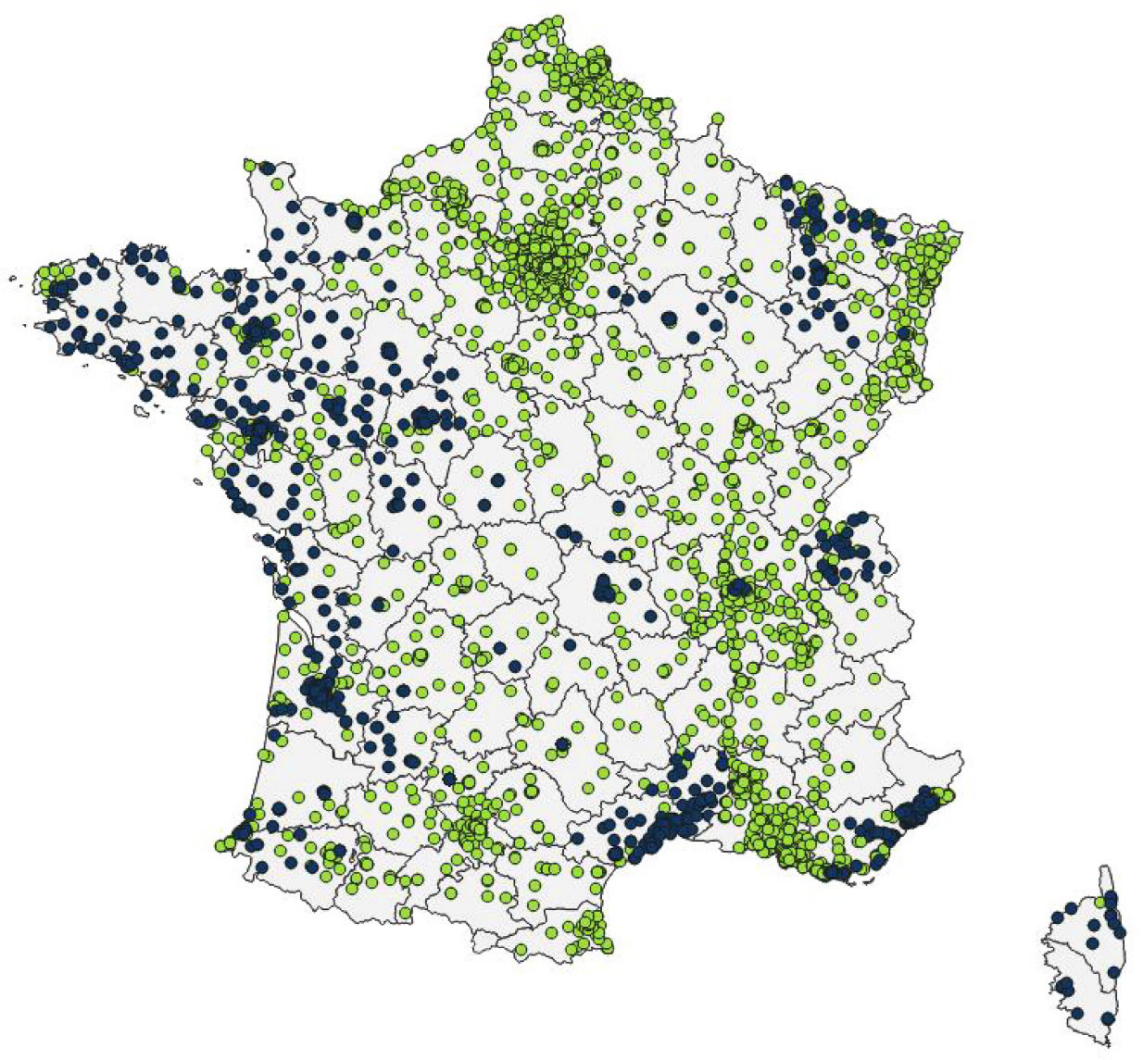
Distribution of the clinical laboratories involved in the MedQual-Ville surveillance (blue dots) among all clinical laboratory sites established in France in 2018 (green dots). Source: Mission PRIMO report 2020.^15^

Despite being a passive surveillance system (i.e. data reporting being initiated by laboratories, rather than health authorities actively searching for information), MedQual-Ville aimed to be representative at a national level. This was an important area for improvement, as participation rates were still limited in some densely populated regions such as Île-de-France (0.1% in 2018) and Hauts-de-France (0% in 2018) compared with others (Corse: 95.8%, Pays de la Loire: 60%, Bretagne: 63.6% in 2018) (Figure 4). Hence, MedQual-Ville results were not nationally representative at this stage (Figures 1 and 3).

Data management was largely automated and highly performant, although the traceability of data validation could slightly be improved (Figures 1 and 2). Comprehensive descriptive data analysis was performed by a multidisciplinary team, which resources and skills appeared sufficient for the current needs of the surveillance system. Dissemination of the results occurred via various channels. Feedback to data providers (clinical laboratories) was delivered in a timely manner via individual quarterly reports (Figure 3). However, it was unknown at this stage whether these reports were actually being read and met the clinical laboratories’ expectations. External communication activities already existed, primarily via annual reports, congresses and the open-access data visualization website MedQual-Ville, where data were displayed in several formats (charts, geographical maps, tables) and raw data could be downloaded freely by the data providers.^17^ Selected AMR indicators were also displayed on the Géodes website of SpF, which has a wider audience.^19^

In the field of evaluation (Figure 1), the MedQual-Ville coordination team already monitored various indicators describing how well the surveillance system was operating (e.g. the proportion of missing data per variable). However, these could not be considered as performance indicators, as no target value and corrective measure (in case of deviation) had been defined at this stage.

## Discussion

### Major strengths and weaknesses

The OASIS evaluation showed that MedQual-Ville was overall a well-performing surveillance system. While the nationwide scope of MedQual-Ville as part of the Mission PRIMO was still recent, it built on a regional network that had long-term experience and strong expertise in AMR surveillance in the community. The additional resources brought by Mission PRIMO made it possible to upgrade existing tools and activities (e.g. data management and visualization tools) and expand them to nationwide surveillance.

Surveillance objectives and procedures were clear to all MedQual-Ville participants. Nonetheless, suggestions were made to consider other possible objectives, such as the development of an early warning system for detection of AMR emergence in collaboration with relevant NRL. Some interviewees also suggested monitoring additional AMR indicators (e.g. the proportion of pan-susceptible strains). Coordination activities were excellent and technical assistance was provided where and when needed. The quality and reliability of the data being produced by the system were very high.

At this stage, the major weakness of the surveillance system was its representativeness. The MedQual-Ville coverage was still largely unbalanced in favour of the west of France, and several densely populated areas were largely uncovered. Of note, participation rates (18.3% in 2018 and 24.9% in 2019) and national coverage had been increasing over recent years. Still, pursuing current efforts to recruit additional laboratories will be critical to achieve national representativeness as required in the terms of reference defined by SpF and the expert committee. Another important area for improvement related to the impact of surveillance activities. While various internal and external communication activities were already being performed, it was not totally clear at this stage how the data and information being generated were made accessible and useful to the end users, especially to local prescribers, and how these data could be translated into concrete action, e.g. contributing to changes in antimicrobial treatment practices.

### Practical recommendations

Practical recommendations for improvement were formulated and classified by order of priority (Table 2). Among high-priority recommendations, MedQual-Ville should strengthen the recruitment of participating laboratories in those regions currently under-represented, hence improving representativeness of national surveillance. It would help to develop incentives to encourage and appreciate the volunteer participation of clinical laboratories, for example via awareness campaigns or the creation of a certification label such as ‘this laboratory contributes to a national initiative of public health interest’. Further promotion of MedQual-Ville at the central level (by the national health authorities and agencies) would also be helpful.

**Table 2.** List of practical recommendations by level of priority

Priority level	Recommendations
High	**Recruitment of clinical laboratories**
	Strengthen the recruitment of participating laboratories in those regions currently under-represented, hence improving representativeness of national surveillance
	Develop incentives to encourage and appreciate the volunteer participation of clinical laboratories. For example, a label ‘this laboratory contributes to a national mission of public health importance’ could be implemented. Continue to acknowledge the volunteer participation of clinical laboratories in every publication of MedQual-Ville
	Further promotion of MedQual-Ville at the central level (by the national health authorities and agencies)
	**Dissemination of the results**
	Strengthen the consideration of MedQual-Ville surveillance data when formulating and updating the national guidelines for good antimicrobial treatment practices
	Engage with regional and local partners (including CPias, regional centres for antimicrobial stewardship and/or clinical laboratories) to further disseminate surveillance results at local level (e.g. to local prescribers)
	Produce a brief two-page summary sheet of key surveillance results to be easily disseminated by regional/local partners to local prescribers and laboratories
	Consider communicating about MedQual-Ville activities in journals dedicated to clinical laboratories and prescribers, as well as the Weekly Epidemiology Bulletin edited by SpF (which has a very broad audience)
	**Performance indicators**
	Define and select performance indicators (max. 10) to monitor key functional and operational aspects of the surveillance system. These should include target values and corrective measures in case of deviation
	Publish performance indicators in the annual activity report of MedQual-Ville, hence facilitating internal and external assessment of surveillance activities
Medium	**Data extraction and submission**
	Consider revising the list of ‘optional’ variables (e.g. patient housing type, sex and age), to encourage clinical laboratories to submit all data of interest to MedQual-Ville
	Wherever possible, encourage laboratories to use automated data extraction from their LIMS, hence improving timeliness and reducing the time/burden of data submission
	**Data visualization**
	Consider adding features to improve data visualization on the MedQual-Ville website, e.g. making it possible to visualize co-resistance patterns
Low	**Get feedback from MedQual-Ville partners**
	Conduct a survey to assess the clinical laboratories’ level of satisfaction with the feedback and information they receive from MedQual-Ville (e.g. quarterly reports)
	** **Conduct a survey among regional partners (e.g. CPias, regional centres for antimicrobial stewardship, regional health agencies) to assess their expectations and level of satisfaction with the information received from the MedQual-Ville
	** **Encourage the participation of clinical laboratories to the annual survey on methods and techniques used; it will help, among others, to monitor their capacities for data extraction and submission, and identify needs for technical assistance
	**Steering committee**
	Consider adding representatives of GPs, clinical laboratories and/or AMR national reference laboratories to the steering committee

Another high-priority recommendation was to improve the dissemination of the results at regional/local level. A two-page summary sheet could be produced and further disseminated to prescribers via regional/local partners (e.g. CPias, regional centres for antimicrobial stewardship and/or clinical laboratories). In addition, the consideration of MedQual-Ville surveillance data when formulating and updating the national guidelines for good antimicrobial treatment practices should be strengthened. These guidelines are later integrated into the ‘Antibioclic’ website, which is the main antimicrobial therapy decision-support tool being used by the French GPs (approx. 9500 connections each day in 2019 and 2020).^22^

It was also recommended to define a set of performance indicators to monitor key functional and operational aspects of the surveillance system. Current indicators covered limited surveillance activities, and could be expanded to other areas, such as the timeliness of data submission and validation, the frequency of use of the MedQual-Ville website, as well as the efforts dedicated to the recruitment of new participating laboratories. These should include target values and corrective measures in case of deviation. It was recommended that these indicators were published in the annual activity report, hence facilitating internal and external assessment of the MedQual-Ville activities. Other medium- and low-priority recommendations related, among others, to data management and visualization, as well as internal evaluation (Table 2). While considered of lower importance, some of the proposed recommendations were fairly simple and could be implemented within short timelines.

### Application of OASIS to human health surveillance

Overall, this study demonstrated that the OASIS framework is applicable to the evaluation of surveillance systems in the human sector. Out of the 20 criteria considered as not applicable, only one of them was because of non-relevance to the human sector. Other reasons related to specific activities and procedures that were absent in MedQual-Ville, including the absence of intermediary units between central and local level (*n *= 7 criteria), the absence of case reporting or notification (*n* = 5) and the absence of active surveillance activities (*n* = 4), as well as other reasons (*n* = 4). Interestingly, a recent application of OASIS to the French surveillance programme for AMR in animal pathogens, an organization similar to MedQual-Ville, also had 22 criteria considered as not applicable.^14^ Still, the evaluation proved helpful to formulate practical recommendations for improvement. Some of these recommendations appeared country-specific, e.g. the necessity to improve national geographic coverage, while others could also be applicable to other countries with similar monitoring systems, e.g. the need to strengthen dissemination of the results towards local/regional actors, hence increasing the value of the information coming from surveillance activities, an aspect which is often overlooked in surveillance systems.^23^ Beyond the recommendations coming from the evaluation, the OASIS tool itself could easily be applied to other countries or systems willing to achieve similar evaluation objectives.

While the OASIS framework covered a large number of aspects related to surveillance functioning, operations and effectiveness, other aspects such as the cost-effectiveness or the level of collaboration of MedQual-Ville with other national programmes for surveillance of AMR or antimicrobial consumption were not addressed. Several frameworks for the evaluation of the level of integration or ‘One Health-ness’ of surveillance systems have recently been developed and assessed;^12^ they appear as complementary tools to evaluation frameworks such as OASIS, and will later be explored to evaluate the level of integration of AMR surveillance in France. Additionally, two new OASIS modules evaluating respectively the cost-effectiveness and the level of collaboration of a surveillance system are currently under development.

Likewise, the relevance and ultimate goal of AMR surveillance in the community when compared with other surveillance activities, e.g. in healthcare facilities, were beyond the scope of this study but have already been addressed elsewhere.^8^ Still, we believe this study provided a useful illustration of how AMR surveillance in the community is currently implemented in France, and could be applied in other countries with similar health systems.

### Conclusions

This work demonstrated that OASIS is a suitable framework for the evaluation of human surveillance systems and that it can easily be adapted to various infectious diseases surveillance systems. We highlighted strengths and limitations of the current AMR surveillance system in the community and nursing homes in France, which has been structured at national level only recently. We strongly recommend such an evaluation to be repeated at a later stage (e.g. in 5 years) in order to monitor progress. There is currently no European programme for AMR surveillance in the community and nursing homes, and no guidance on how such a surveillance should be implemented. Hence, the present study contributes to filling this gap by performing an evaluation of the ongoing surveillance system in France and formulating recommendations on how this programme could be improved in the future.

## Supplementary Material

dlac078_Supplementary_Data

## Supplementary data

Table S1 is available as Supplementary data at *JAC-AMR* Online.

## References

[1] Cassini A, Högberg LD, Plachouras D et al Attributable deaths and disability-adjusted life-years caused by infections with antibiotic-resistant bacteria in the EU and the European Economic Area in 2015: a population-level modelling analysis. Lancet Infect Dis 2019; 19: 56–66. 10.1016/S1473-3099(18)30605-4 30409683 PMC6300481

[2] Touat M, Opatowski M, Brun-Buisson C et al A payer perspective of the hospital inpatient additional care costs of antimicrobial resistance in France: a matched case–control study. Appl Health Econ Health Policy 2019; 17: 381–9. 10.1007/s40258-018-0451-1 30506456 PMC6535148

[3] European Commission . A European One Health Action Plan Against Antimicrobial Resistance (AMR). 2017. https://ec.europa.eu/health/system/files/2020-01/amr_2017_action-plan_0.pdf.

[4] CPias Pays de la Loire . Centre d’appui pour la Prévention des Infections Associées aux Soins du Pays de la Loire . https://www.cpias-pdl.com/.

[5] Thibaut S, Coeffic T, Boutoille D et al A multi-year decline of multidrug-resistant Escherichia coli in French nursing homes and primary care: are we on the good track? ECCMID, 2020. Abstract 6376. https://www.escmid.org/escmid_publications/eccmid_abstract_book/.

[6] Thibaut S, Caillon J, Huart C et al Susceptibility to the main antibiotics of Escherichia coli and Staphylococcus aureus strains identified in community acquired infections in France (MedQual, 2004-2007). Med Mal Infect 2010; 40: 74–80. 10.1016/j.medmal.2009.01.011 19837526

[7] Larramendy S, Gaultier A, Giffon S et al Prevalence of extended-spectrum β-lactamase-producing *Escherichia coli* in community-acquired urinary tract infections in Western France. Med Mal Infect 2020; 50: 297–300. 10.1016/j.medmal.2019.09.009 31575447

[8] Chin TL, McNulty C, Beck C et al Antimicrobial resistance surveillance in urinary tract infections in primary care. J Antimicrob Chemother 2016; 71: 2723–8. 10.1093/jac/dkw223 27353470

[9] Rempel OR, Laupland KB. Surveillance for antimicrobial resistant organisms: potential sources and magnitude of bias. Epidemiol Infect 2009; 137: 1665–73. 10.1017/S0950268809990100 19493372

[10] Cornaglia G, Hryniewicz W, Jarlier V et al European recommendations for antimicrobial resistance surveillance. Clin Microbiol Infect 2004; 10: 349–83. 10.1111/j.1198-743X.2004.00887.x 15059129

[11] Calba C, Goutard FL, Hoinville L et al Surveillance systems evaluation: a systematic review of the existing approaches. BMC Public Health 2015; 15: 448. 10.1186/s12889-015-1791-5 25928645 PMC4418053

[12] Sandberg M, Hesp A, Aenishaenslin C et al Assessment of evaluation tools for integrated surveillance of antimicrobial use and resistance based on selected case studies. Front Vet Sci 2021; 8: 620998. 10.3389/fvets.2021.620998 34307513 PMC8298032

[13] Hendrikx P, Gay E, Chazel M et al OASIS: an assessment tool of epidemiological surveillance systems in animal health and food safety. Epidemiol Infect 2011; 139: 1486–96. 10.1017/S0950268811000161 21385516

[14] Mader R, Jarrige N, Haenni M et al OASIS evaluation of the French surveillance network for antimicrobial resistance in diseased animals (RESAPATH): success factors underpinning a well-performing voluntary system. Epidemiol Infect 2021; 149: e104. 10.1017/S0950268821000856 33877045 PMC8161364

[15] Mission PRIMO . Surveillance de la Résistance Bactérienne aux Antibiotiques en Soins de Ville et en Établissements pour Personnes Âgées Dépendantes. Réseau Primo: Résultats 2018. Santé Publique France, 2020.

[16] Mission PRIMO . Surveillance de la Résistance Bactérienne aux Antibiotiques en Soins de Ville et en Établissements pour Personnes Âgées Dépendantes. Réseau Primo: Résultats 2019. 2021. https://www.santepubliquefrance.fr/import/surveillance-de-la-resistance-bacterienne-aux-antibiotiques-en-soins-de-ville-et-en-etablissements-pour-personnes-agees-dependantes.-reseau-primo.

[17] Mission PRIMO . MedQual-Ville. 2020. https://medqualville.antibioresistance.fr/.

[18] Jehl F, Bonnet R, Bru J-P et al Comité de l’Antibiogramme de la Société Française de Microbiologie. Recommandations 2019V.2.0 Mai 2019. Société Française de Microbiologie, 2019.

[19] Santé Publique France. Géodes—Géodonnées en Santé Publique. 2021. https://geodes.santepubliquefrance.fr.

[20] German RR, Lee LM, Horan JM et al Updated guidelines for evaluating public health surveillance systems: recommendations from the guidelines working group. MMWR Morb Mortal Wkly Rep 2001; 50: 1–35. 18634202

[21] WHO . Protocol for the Assessment of National Communicable Disease Surveillance and Response Systems: Guidelines for Assessment Teams. 2001. https://apps.who.int/iris/handle/10665/66787.

[22] Université Paris Diderot Département de Médecine Générale . Antibioclic: Antibiothéraphie Rationnelle en Soins Primaires. https://antibioclic.com/.

[23] Antoine-Moussiaux N, Vandenberg O, Kozlakidis Z et al Valuing health surveillance as an information system: interdisciplinary insights. Front Public Health 2019; 7: 138. 31263687 10.3389/fpubh.2019.00138 PMC6585471

